# Protective Effects of Bee Venom-Derived Phospholipase A_2_ against Cholestatic Liver Disease in Mice

**DOI:** 10.3390/biomedicines9080992

**Published:** 2021-08-11

**Authors:** Jung-Yeon Kim, Hyo-Jeong Jang, Jaechan Leem, Gyun-Moo Kim

**Affiliations:** 1 Department of Immunology, School of Medicine, Catholic University of Daegu, Daegu 42472, Korea; jy1118@cu.ac.kr; 2 Department of Pediatrics, School of Medicine, Keimyung University, Daegu 42601, Korea; bearinspring@hotmail.com; 3 Department of Emergency Medicine, School of Medicine, Catholic University of Daegu, Daegu 42472, Korea

**Keywords:** bee venom, phospholipase A2, cholestatic liver disease, apoptosis, inflammation, fibrosis

## Abstract

Hepatocyte apoptosis and inflammation play important roles in cholestatic liver diseases. Bee venom-derived secretory phospholipase A2 (bvPLA2) has been shown to ameliorate various inflammatory diseases. However, whether bvPLA2 has a therapeutic effect against cholestatic liver disease has not been evaluated. Therefore, we investigated the effects of bvPLA2 on cholestatic liver injury and fibrosis in a murine model of 3,5-diethoxycarbonyl-1,4-dihydrocollidine (DDC) diet feeding. The administration of bvPLA2 ameliorated liver damage, cholestasis, and fibrosis in DDC diet-fed mice, as assessed by serum biochemical tests and histological examinations. In addition, bvPLA2 reduced myofibroblast accumulation, concomitant with suppression of transforming growth factor-β signaling cascade. The administration of bvPLA2 inhibited hepatocyte apoptosis in DDC diet-fed mice as represented by a reduction in the number of cells stained with terminal deoxynucleotidyl transferase-mediated dUTP nick-end labeling and suppression of caspase-3 activation. Moreover, bvPLA2 reduced cytokine production along with the inhibition of the nuclear factor kappa-B pathway. The number of regulatory T-cells was increased by bvPLA2, while the number of other immune cells, including neutrophils, macrophages, and CD8^+^ T-cells, was decreased. Our data indicate that the administration of bvPLA2 ameliorates cholestatic liver injury and fibrosis by inhibiting hepatocyte apoptosis and inflammation.

## 1. Introduction

Cholestatic liver diseases such as primary sclerosing cholangitis and primary biliary cholangitis are a significant cause of liver-related death and the leading indication for liver transplantation in pediatric patients [1,2]. Given that treatment options for these diseases remain limited [2], the development of novel pharmacologic treatments is essential to improving the health outcomes of patients with the diseases. Cholestasis is defined as an interruption in bile flow due to the obstruction of bile ducts or impaired secretion by hepatocytes [2]. In cholestatic liver diseases, cholestasis induces hepatic injury and fibrosis [3]. Although the exact mechanisms are not yet fully understood, apoptotic cell death and inflammation are critically involved in cholestatic liver diseases [3,4].

Bee venom therapy has long been used for the therapy of various human diseases, especially in Asia [5,6]. Bee venom is a complex mixture of peptides, enzymes, and other bioactive components. Among them, phospholipase A2 (PLA2) derived from bee venom belongs to the group III secretory PLA2 (sPLA2) enzymes and constitutes 10–12% of dry bee venom [7]. PLA2 preferentially cleaves gycerophospholipids at the sn-2 position, liberating fatty acids and lysophospholipids. Although many of the mechanisms of action still remain unclear, accumulating evidence suggests that bee venom-derived sPLA2 (bvPLA2) exerts several beneficial actions, including anti-bacterial, anti-tumor, anti-neuronal injury and anti-nociceptive effects [7]. Moreover, it has been shown that the administration of bvPLA2 ameliorated a variety of inflammatory diseases in rodents, such as acute kidney injury [8], acute lung inflammation [9], atherosclerosis [10], atopic dermatitis [11], and allergic asthma [12]. In addition, mice injected with bvPLA2 have been reported to exhibit less liver damage after the administration of high-dose acetaminophen compared to control mice [13]. However, whether bvPLA2 has a therapeutic effect against cholestatic liver injury has not been investigated.

The 3,5-diethoxycarbonyl-1,4-dihydrocollidine (DDC) diet model is a widely used animal model for cholestatic liver diseases [14,15]. DDC is a porphyrinogenic agent and strong inducer of δ-aminolevulinate synthetase, the rate-limiting enzyme of heme biosynthesis. Feeding rodents a DDC-enriched diet increases the secretion of hepatotoxic protoporphyrins, along with the formation of protoporhyrin plugs [15]. The latter leads to obstruction of small bile ducts that initiate cholestasis. This animal model reproduces the major histopathological hallmarks of human cholestatic liver disease, such as remodeling biliary compartments, periductular fibrosis, and inflammatory cell infiltration [14]. In the present study, we evaluated the effects of bvPLA2 on DDC diet-induced cholestatic liver injury. In addition, the underlying mechanisms were also investigated.

## 2. Materials and Methods

### 2.1. Animal Study Protocol

All animal care and experimental protocols were performed in accordance with the Institutional Animal Care and Use Committee of the Daegu Catholic University Medical Center (Approval number: DCIAFCR-210112-19-Y, approval date: 12 January 2021). Male seven-week-old C57BL/6N mice were obtained from HyoSung Science Inc. (Daegu, Korea) and kept on a 12-h light/dark cycle at 20–24 °C and 60–70% humidity. After one week, the mice were arbitrarily divided into three groups (n = 8) as follows: (a) vehicle-treated control group (Veh): C57BL/6N mice were fed with a normal chow diet for four weeks; (b) DDC diet-fed group (DDC): C57BL/6N mice were fed with a 0.1% DDC diet for four weeks; (c) DDC diet-fed group treated with bvPLA2 (DDC+bvPLA2): C57BL/6N mice were fed with a 0.1% DDC diet and were intraperitoneally injected with bvPLA2 (0.2 mg/kg; dissolved in phosphate-buffered saline; Sigma-Aldrich, St. Louis, MO, USA) twice a week for four weeks. The DDD diet was acquired from RaonBio Inc. (Yongin, Korea). The Veh group and the DDC group received an intraperitoneal injection with an equal volume of the vehicle twice a week for four weeks. The dose of bvPLA2 was chosen based on previous studies [8,13]. All mice were sacrificed after four weeks of treatment. Blood samples and liver tissues were rapidly collected for further analysis.

### 2.2. Biochemical Analysis

Levels of serum aspartate aminotransferase (AST), alanine aminotransferase (ALT), alkaline phosphatase (ALP), and total bilirubin were analyzed using a 7020 automatic analyzer (Hitachi, Osaka, Japan). Serum interleukin-10 (IL-10) levels were measured using the Mouse IL-10 Quantikine ELISA Kit (R&D Systems, Minneapolis, MN, USA) according to the manufacturer’s instructions.

### 2.3. Histological Analysis, Immunohistochemistry, and Immunofluorescent Staining

Isolated liver tissues were fixed in 10% formalin and dehydrated. After dehydration, the tissues were cleared and embedded in paraffin. Paraffin sections of 4-μm thickness were mounted on a microscope slide and stained with hematoxylin & eosin (H&E) or Masson’s trichrome stain. 

For immunohistochemical staining, the sections were incubated with one of the following primary antibodies: anti-collagen I, anti-fibronectin, anti-F4/80, or anti-CD8. These antibodies were purchased from Abcam (Cambridge, UK) except for F4/80 (Santa Cruz Biotechnology Inc., Dallas, TX, USA). After washing, the sections were probed with a secondary antibody. Images were captured using a confocal microscope (Nikon, Tokyo, Japan). The percentage of positively stained area was analyzed in five randomly selected fields per liver sample with i-Solution DT software (IMT i-Solution Inc., Coquitlam, BC, Canada). The number of F4/80 or CD4-positive cells was counted in five randomly selected fields per liver sample.

For immunofluorescent staining, liver sections were blocked in phosphate-buffered saline with 5% bovine serum albumin. The sections were proved with one of the following primary antibodies: anti-cytokeratin 19 (CK19; Abcam), anti-α-smooth muscle actin (α-SMA; Sigma-Aldrich), anti-forkhead box protein P3 (Foxp3; Santa Cruz Biotechnology Inc.), or anti-Ly6B.2 (Abcam). After washing, the sections were incubated with a secondary antibody conjugated with Alexa Fluor 488 or Alexa Fluor 555 (Invitrogen, Carlsbad, CA, USA). For nuclear counterstaining, 4′, 6-diamidino-2-phenylindole (DAPI) was used. The percentage of CK19-stained area was analyzed in five randomly selected fields per liver sample. The number of positively stained cells counted in five randomly selected fields per liver sample.

### 2.4. Western Blot Analysis

Proteins were extracted from liver tissues with a lysis buffer (Sigma-Aldrich). Protein samples were separated by sodium dodecyl sulfate-polyacrylamide gel electrophoresis and then transferred to a nitrocellulose membrane. The membranes were incubated with one of the following primary antibodies: anti-cleaved caspase-3 (1:1000; Cell Signaling, Danvers, MA, USA), anti-poly(ADP-ribose) polymerase-1 (PARP-1; 1:1000; Cell Signaling) anti-tumor necrosis factor-α (TNF-α; 1:1000; Abcam), anti-IL-6 (1:1000; Abcam), anti-IκBα (1:1000; Cell Signaling), anti-p-IκBα (1:1000; Cell Signaling), anti-nuclear factor-κB (NF-κB) p65 (1:1000; Cell Signaling), anti-p-NF-κB p65 (1:1000; Cell Signaling), anti-transforming growth factor-β1 (TGF-β1; 1:1000; Abcam), anti-Smad2/3 (1:1000; Cell Signaling), anti-p-Smad2/3 (1:1000; Cell Signaling), anti-glyceraldehyde-3-phosphate dehydrogenase (GAPDH; 1:3000; Cell Signaling). The membranes were washed and probed with a secondary antibody conjugated with horseradish peroxidase. The signal intensities were analyzed using the iBright™ CL1500 Imaging System (Thermo Fisher Scientific, Waltham, MA, USA).

### 2.5. TdT-Mediated dUTP Nick End Labeling (TUNEL) Assay

TUNEL staining of liver sections was performed using a TUNEL assay kit (Roche Diagnostics, Indianapolis, IN, USA) according to the manufacturer′s instructions. Counterstaining was performed with DAPI. The number of TUNEL-positive cells was counted in five randomly selected fields per liver sample.

### 2.6. Statistical Analysis

Data are presented as mean ± standard error of the mean (SEM). Statistical analyses were performed using the one-way analysis of variance (ANOVA) with Bonferroni’s post hoc tests. A *p*-value less than 0.05 was considered to represent statistical significance.

## 3. Results

### 3.1. Administration of bvPLA2 Ameliorated Cholestatic Liver Injury and Fibrosis in DDC Diet-Fed Mice

**Figure 1:**
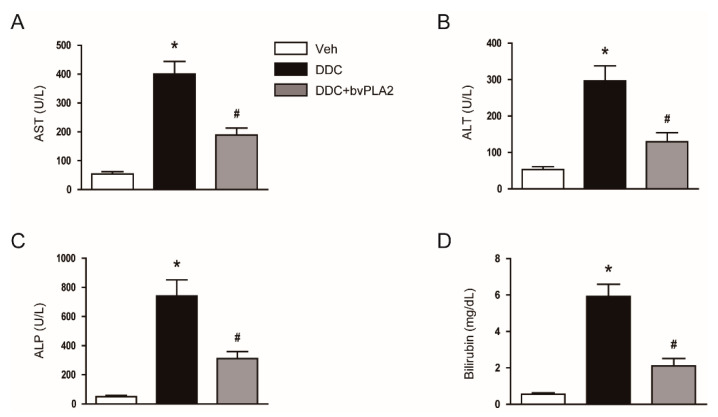
Biochemical markers of liver injury and cholestasis in all study groups. C57BL/6N mice were fed with a diet containing 0.1% 3,5-diethoxycarbonyl-1,4-dihydrocollidine (DDC) and were intraperitoneally injected with bee venom-derived secretory phospholipase A2 (bvPLA2; 0.2mg/kg) twice a week for four weeks. (**A**) Serum aspartate aminotransferase (AST) levels. (**B**) Serum alanine aminotransferase (ALT) levels. (**C**) Serum alkaline phosphatase (ALP) levels. (**D**) Serum total bilirubin levels. n = 8 per group. * *p* < 0.05 vs. the vehicle-treated control group (Veh). ^#^ *p* < 0.05 vs. the DDC diet-fed group (DDC).

Feeding mice a 0.1% DDC diet for four weeks led to a marked increase in serum levels of AST (Figure 1A) and ALT (Figure 1B), indicators of hepatocyte injury, in mice. Serum markers of cholestasis, ALP (Figure 1C) and total bilirubin (Figure 1D), were also highly increased after feeding with a DDC diet. However, these changes in serum markers were significantly attenuated by the administration of bvPLA2 (Figure 1A–D).

**Figure 2:**
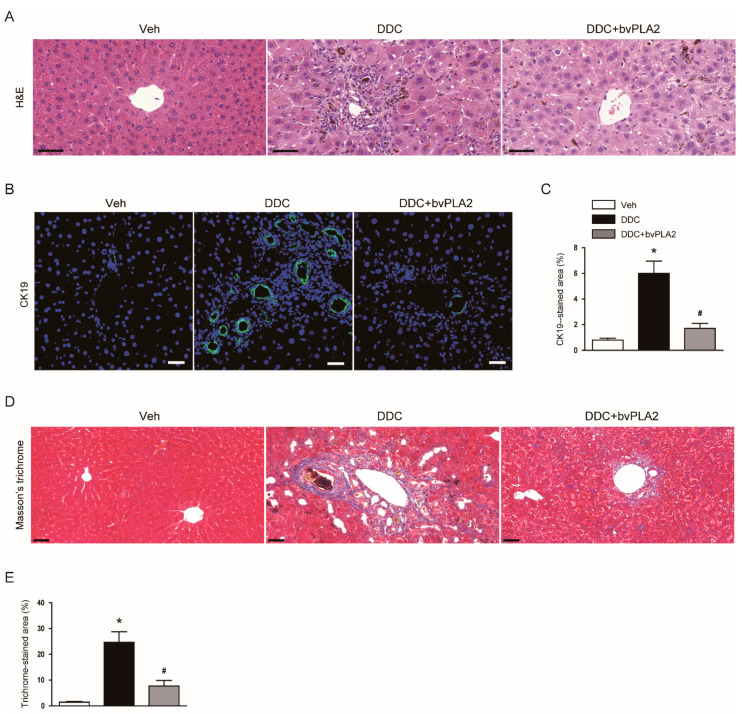
Histological alterations of the livers in all study groups. (**A**) Hematoxylin and eosin (H&E) staining of liver tissues. Scale bar = 50 μm. (**B**) Immunofluorescent staining of liver tissues for cytokeratin 19 (CK19). Scale bar = 40 μm. (**C**) Percentage of area stained with anti-CK19 antibody. (**D**) Masson’s trichrome staining of liver tissues. Scale bar = 50 μm. (**E**) Percentage of area stained with Masson’s trichrome stain. n = 8 per group. * *p* < 0.05 vs. Veh. ^#^ *p* < 0.05 vs. DDC.

We observed that DDC diet-fed mice displayed histological alterations such as deposition of pigment plugs in small bile ducts and inflammatory cell infiltration, as shown by hematoxylin & eosin staining of liver tissues (Figure 2A). Immunofluorescent staining for CK19, a marker of biliary epithelial cells [16,17], showed that CK19-stained area was increased after DDC feeding in livers (Figure 2B,C), indicating that DDC feeding induced ductular reaction. DDC feeding also resulted in marked fibrosis in livers, as represented by an increase in area stained by Masson’s trichrome stain (Figure 2D,E). However, these abnormalities were significantly alleviated by bvPLA2 (Figure 2A–E).

### 3.2. Administration of bvPLA2 Inhibited Expression of Extracellular Matrix Proteins, Myofibroblast Accumulation, and TGF-β Signaling Pathway in DDC Diet-Fed Mice

**Figure 3:**
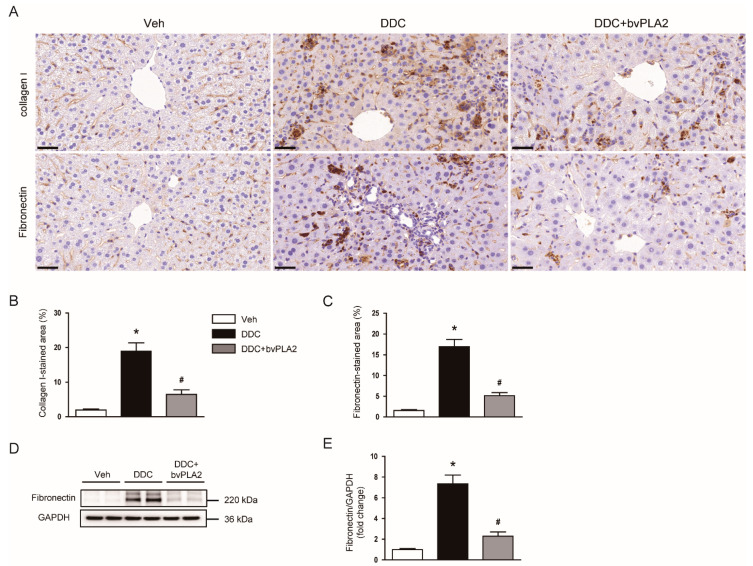
Effect of bvPLA2 on expression of extracellular matrix proteins in DDC diet-fed mice. (**A**) Immunohistochemical staining of liver tissues for collagen I or fibronectin. Scale bar = 100 μm. (**B**) Percentage of area stained with anti-collagen I antibody. (**C**) Percentage of area stained with anti-fibronectin antibody. (**D**) Western blotting of fibronectin in liver tissues. (**E**) Quantification of western blots for fibronectin. n = 8 per group. * *p* < 0.05 vs. Veh. ^#^ *p* < 0.05 vs. DDC.

To further investigate the role of bvPLA2 in the DDC diet-induced fibrosis, we next examined its effect on the expression of extracellular matrix proteins. Immunohistochemical staining showed that DDC feeding largely increased the percentage of area stained with collagen I or fibronectin (Figure 3A–C). Western blot analysis also confirmed the elevated levels of fibronectin protein in livers of DDC diet-fed mice (Figure 3D,E). However, the administration of bvPLA2 significantly attenuated the expression of extracellular matrix proteins (Figure 3A–E).

**Figure 4:**
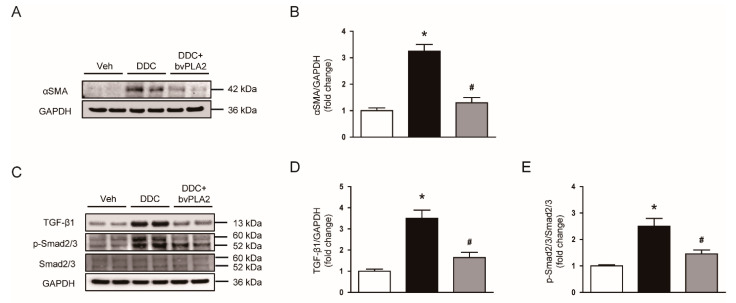
Effect of bvPLA2 on α-smooth muscle actin (α-SMA) expression and transforming growth factor-β1 (TGF-β1) signaling cascade in DDC diet-fed mice. (**A**) Western blotting of α-SMA in liver tissues. (**B**) Quantification of western blot for α-SMA. (**C**) Western blotting of TGF-β1 and p-Smad2/3 in liver tissues. (**D**) Quantification of western blot for TGF-β1 (**E**) Quantification of western blot for p-Smad2/3. n = 8 per group. * *p* < 0.05 vs. Veh. ^#^ *p* < 0.05 vs. DDC.

Myofibroblasts are the primary cells responsible for the production of extracellular matrix proteins [18,19]. We next examined the effect of bvPLA2 on protein levels of α-SMA, a marker of myofibroblasts [20]. DDC feeding led to an increase in α-SMA expression, which was significantly reduced by bvPLA2 (Figure 4A,B). These results indicate that bvPLA2 decreased the accumulation of myofibroblasts in liver tissues.

TGF-β/Smad2/3 signaling pathway plays a major role in the expression of fibrosis-related genes [21]. We found that increased protein levels of TGF-β1 and phosphorylated Smad2/3 after DDC feeding were significantly reduced by bvPLA2 (Figure 4C–E). These results suggest that suppression of TGF-β/Smad2/3 signaling pathway by bvPLA2, at least in part, contributes to its inhibitory effect on fibrosis.

### 3.3. Administration of bvPLA2 Attenuated Apoptotic Cell Death in DDC Diet-Fed Mice

**Figure 5:**
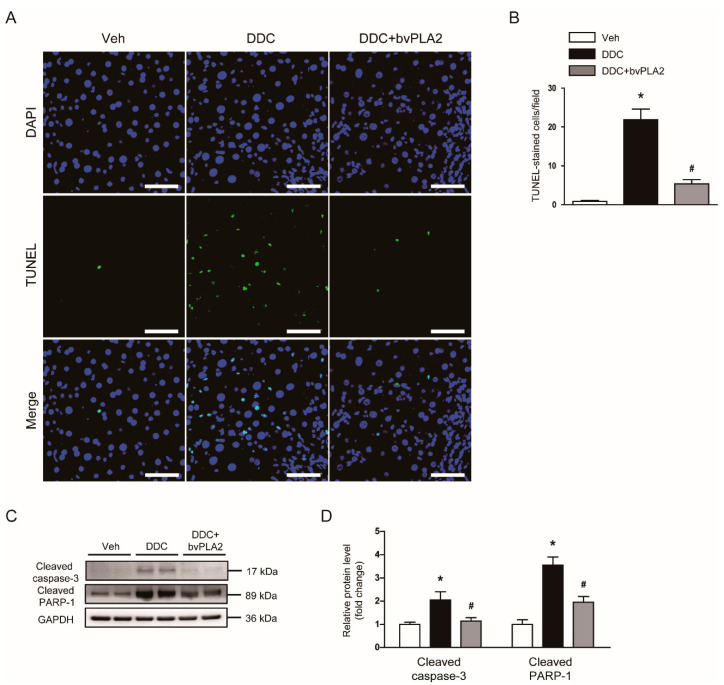
Effect of bvPLA2 on apoptotic cell death in DDC diet-fed mice. (**A**) TdT-mediated dUTP nick end labeling (TUNEL) assay on liver tissues. Scale bar = 40 μm. (**B**) Number of TUNEL-stained cells. (**C**) Western blotting of cleaved caspase-3 and cleaved poly(ADP-ribose) polymerase-1 (PARP-1) in liver tissues. (**D**) Quantification of western blots for cleaved caspase-3 and cleaved PARP-1. n = 8 per group. * *p* < 0.05 vs. Veh. ^#^ *p* < 0.05 vs. DDC.

It has been known that hepatocyte apoptosis plays a crucial role in the progression of cholestatic liver disease, contributing to tissue fibrosis [4]. Thus, we next examined the effect of bvPLA2 on hepatocyte apoptosis in DDC diet-fed mice. TUNEL assay revealed that DDC feeding largely increased the number of apoptotic cells in livers (Figure 5A,B). Protein levels of cleaved caspase-3 and cleaved PARP-1 were also increased after DDC feeding (Figure 5C,D). However, the DDC diet-induced apoptosis was significantly alleviated by bvPLA2 (Figure 5A–D).

### 3.4. Administration of bvPLA2 Inhibited Cytokine Production in DDC Diet-Fed Mice

**Figure 6:**
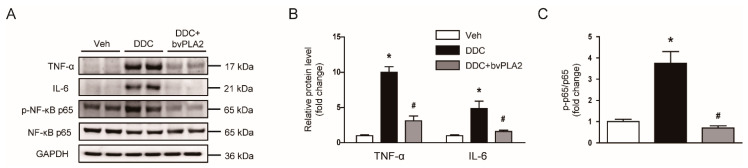
Effect of bvPLA2 on cytokine production and nuclear factor-κB (NF-κB) signaling pathway in DDC diet-fed mice. (**A**) Western blotting of tumor necrosis factor-α (TNF-α), interleukin-6 (IL-6), and p-nuclear factor-κB (NF-κB) p65 in liver tissues. (**B**) Quantification of western blots for TNF-α and IL-6. (**C**) Quantification of western blot for p-NF-κB p65. n = 8 per group. * *p* < 0.05 vs. Veh. ^#^ *p* < 0.05 vs. DDC.

Inflammation is also critically involved in the pathogenesis of cholestatic liver disease [3]. Therefore, we next evaluated the effect of bvPLA2 on cytokine production in livers. We found that protein levels of TNF-α and IL-6 were increased after DDC feeding (Figure 6A,B). Administration of bvPLA2 significantly reduced the amounts of these cytokines (Figure 6A,B). Increased levels of phosphorylated forms of NF-κB p65 after DDC feeding were also significantly decreased by bvPLA2 (Figure 6A,C). These results suggest that bvPLA2 inhibited cytokine production, concomitant with suppression of NF-κB pathway.

### 3.5. Administration of bvPLA2 Modulated Immune Cell Infiltration in DDC Diet-Fed Mice

**Figure 7:**
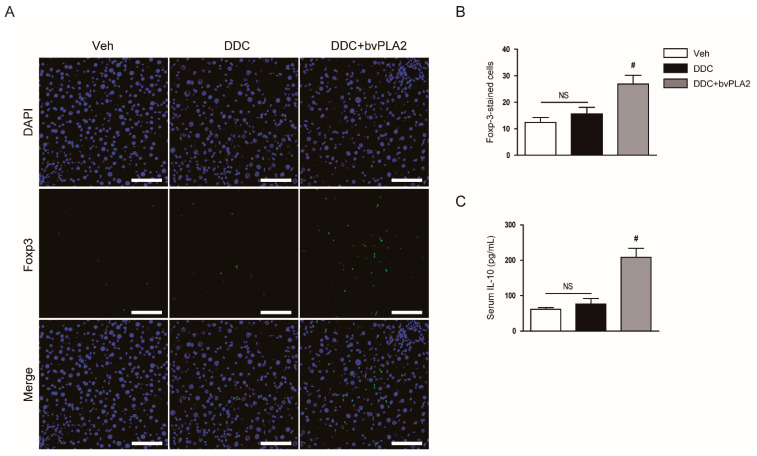
Effect of bvPLA2 on regulatory T-cells in DDC diet-fed mice. (**A**) Immunofluorescent staining of liver tissues for forkhead box protein P3 (Foxp3). Scale bar = 50 μm. **(B**) Number of Foxp3-stained cells. (**C**) Serum interleukin-10 (IL-10) levels. n = 8 per group. ^#^ *p* < 0.05 vs. DDC. NS, not significant.

Regulatory T (Treg) cells are a specialized subset of CD4^+^ T-cells that express the Treg-specific transcription factor Foxp3 and play major roles in suppressing excessive immune responses through secreting immunosuppressive cytokines such as IL-10 [22]. Previous studies have shown that bvPLA2 suppressed inflammation and tissue injury by increasing the Treg population [8,9,10]. Therefore, we next investigated whether bvPLA2 regulates the accumulation of Treg cells in livers of DDC diet-fed mice. To this end, immunohistochemical staining of liver tissues for Foxp3 was performed. Administration of bvPLA2 significantly increased the number of Foxp3-positive cells (Figure 7A,B). Serum IL-10 levels were also elevated in bvPLA2-treated mice compared to control mice or DDC diet-fed mice (Figure 7C). These results suggest that bvPLA2 increased the Treg population in DDC diet-fed mice.

**Figure 8:**
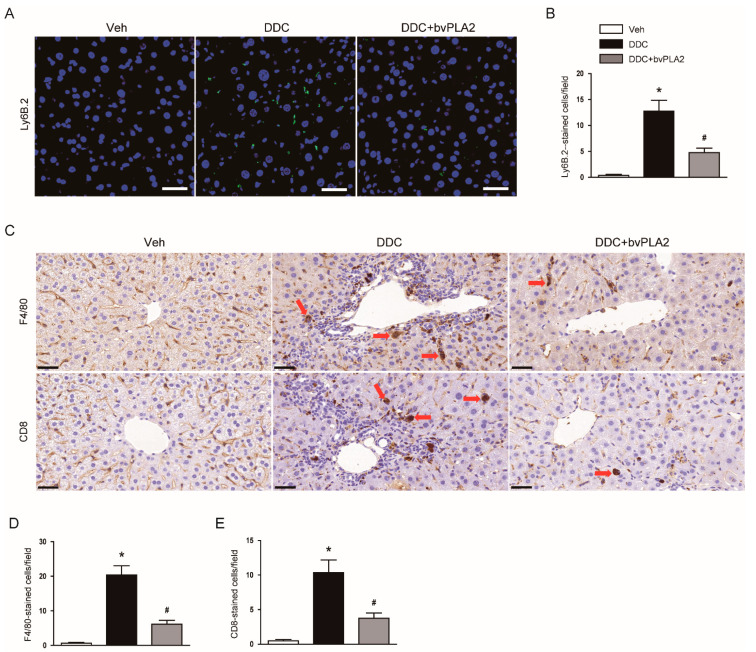
Effect of bvPLA2 on the accumulation of pro-inflammatory cells in DDC diet-fed mice. (**A**) Immunofluorescent staining of liver tissues for Ly6B.2. Scale bar = 40 μm. (**B**) Number of Ly6B.2-stained cells. (**C**) Immunohistochemical staining of liver tissues for F4/80 or CD8. Red arrows indicate positively stained cells. Scale bar = 100 μm. (**D**) Number of F4/80-stained cells. (**E**) Number of CD8-stained cells. n = 8 per group. * *p* < 0.05 vs. Veh. ^#^ *p* < 0.05 vs. DDC.

It has also been shown that pro-inflammatory cells were infiltrated into the liver in DDC diet-fed mice, promoting inflammatory responses [23,24]. The number of Ly6B.2^+^ neutrophils was increased after DDC feeding, as represented by immunofluorescent staining (Figure 8A,B). DDC feeding also increased the number of F4/80^+^ macrophages and CD4^+^ T-cells (Figure 8C–E). However, bvPLA2 attenuated the accumulation of these pro-inflammatory cells in livers (Figure 8A–E).

## 4. Discussion

Cholestatic liver diseases are associated with a significant morbidity and mortality in infants and children [1,2], but medical therapies are limited. Emerging evidence suggests that bvPLA2 has a therapeutic effect against various inflammatory diseases [8,9,10,11,12,13]. In this study, we aimed to investigate the potential effect of bvPLA2 against cholestatic liver disease. We demonstrated that administration of bvPLA2 ameliorated the DDC diet-induced cholestatic liver injury and fibrosis through suppressing apoptosis and inflammatory responses.

In cholestatic liver diseases, both functional impairment and structural obstruction of bile ducts result in the accumulation of bile acids in serum and in hepatocytes, leading to hepatocyte damage [3]. DDC feeding resulted in cholestasis and hepatocyte damage, as represented by elevated serum levels of AST, ALT, ALP, and total bilirubin. However, administration of bvPLA2 significantly inhibited the DDC diet-induced cholestasis and liver injury. Furthermore, bvPLA2 attenuated histological abnormalities such as deposition of pigment plugs in small bile ducts and inflammatory cell infiltration. The DDC diet-induced fibrosis was also significantly alleviated by bvPLA2. It has been shown that bvPLA2 protected against acetaminophen-induced acute liver injury in mice [13]. In addition, a recent study reported that bvPLA2 attenuated obesity-associated hepatotoxicity in high fat diet-induced obese mice [25]. Taken together, these results suggest that bvPLA2 may have therapeutic effects not only in acute liver injury, but also chronic liver disease.

The ductular reaction is characterized by the proliferation of biliary epithelial cells induced by liver injuries and is often observed in patients with cholestatic liver diseases [26]. Proliferating biliary epithelial cells secrete various profibrogenic cytokines to activate hepatic stellate cells, promoting the production of extracellular matrix proteins [16,27]. As previously reported [16,17], DDC feeding induced ductular reaction, as shown by an increase in area stained by an antibody against CK19, a marker of biliary epithelial cells. Administration of bvPLA2 significantly reduced the DDC diet-induced ductular reaction. Thus, the reduced ductular reaction induced by bvPLA2 may contribute to the amelioration of cholestatic liver fibrosis.

Myofibroblasts are the main cells responsible for the production and secretion of extracellular matrix proteins [18,19]. Previously, it was reported that DDC feeding largely increased the number of α-SMA-positive myofibroblasts in livers [28]. In the present study, protein levels of α-SMA were increased after DDC feeding, which was significantly attenuated by bvPLA2. These findings suggest that suppression of myofibroblasts by bvPLA2 treatment is critically involved, at least in part, in its anti-fibrotic action. It has been well known that TGF-β1 is a key player in tissue fibrosis [21]. Binding of TGF-β1 to its receptors induces phosphorylation of Smad2 and Smad3. Activated Smad2/3 bind to Smad4, forming a heteromeric complex, which translocates into the nucleus. This cascade modulates the expression of fibrosis-associated genes [21]. Administration of bvPLA2 significantly alleviated TGF-β1 expression and Smad2/3 activation in DDC diet-fed mice. Expression of extracellular matrix proteins, such as collagen I and fibronectin, was decreased by bvPLA2. The TGF-β/Smad pathway also plays an essential role in myofibroblast differentiation and activation [18]. Therefore, our findings suggest that bvPLA2 ameliorated myofibroblast accumulation and fibrotic changes induced by DDC feeding through inhibiting TGF-β/Smad pathway.

Hepatocyte apoptosis plays a critical role in liver fibrosis [4]. In cholestatic liver disease, bile acid-induced apoptosis is considered a main cause of hepatocyte damage [29]. Thus, to investigate the underlying mechanisms for the therapeutic effects of bvPLA2 against fibrosis, we performed TUNEL staining to detect apoptotic hepatocytes. DDC feeding increased the number of TUNEL-positive cells and activated caspase-3 pathway in livers. However, administration of bvPLA2 significantly reduced the DDC diet-induced apoptosis. Because bvPLA2 inhibited cholestasis, suppression of cholestasis by bvPLA2 may contribute, at least partially, to its anti-apoptotic effect. In addition, previous studies also suggest a direct anti-apoptotic effect of bee venom or bvPLA2 on several types of cells, such as hepatocytes and neuron cells [30,31,32].

Besides hepatocyte apoptosis, inflammation is also an important contributor to the development of cholestatic liver disease [3]. Previous studies have reported that the expression of several inflammatory cytokines was largely elevated after DDC feeding [23,24]. The administration of bvPLA2 significantly decreased hepatic levels of TNF-α and IL-6. NF-κB signaling cascade was also inhibited by bvPLA2. Consistent with our findings, recent studies have shown that bvPLA2 decreased cytokine production in several inflammatory diseases such as acute lung inflammation [9], atherosclerosis [10], and allergic asthma [12] in rodents.

Treg cells play a key role in the suppression of excessive immune responses and maintenance of immune tolerance [22]. Some of their immunosuppressive functions are mediated by the secretion of inhibitory cytokines such as IL-10. Accumulating evidence suggests that bvPLA2 increases the Treg population to inhibit inflammation and tissue injury [8,9,10]. It has been shown that CD206 receptor expressed in dendritic cell membranes plays an important role in the expansion of Treg cells induced by bvPLA2 [8]. In this study, we found that administration of bvPLA2 increased the number of Foxp3^+^ Treg cells and serum IL-10 levels in DDC diet-fed mice. These results suggest that bvPLA2-induced expansion of Treg cells may contribute to the suppression of liver inflammation and fibrosis. Recent studies have shown that Treg cells inhibit liver inflammation and fibrosis in animal models of liver fibrosis induced by carbon tetrachloride [33] or bile duct ligation [34]. Therefore, an increase in Treg population after bvPLA2 treatment may be mainly responsible for the suppression of liver inflammation and fibrosis. Administration of bvPLA2 also suppressed the accumulation of Ly6B.2^+^ neutrophils, F4/80^+^ macrophages, and CD4^+^ T-cells in DDC diet-fed mice. It has been known that Treg cells are able to inhibit the activation and migration of other pro-inflammatory cells [22]. In an animal model of cholestatic liver disease, infiltration of CD8^+^ T-cells was enhanced by depletion of Foxp3^+^ Treg cells [34]. Altogether, these results suggest that bvPLA2 suppressed the accumulation of pro-inflammatory cells in fibrotic livers through increasing the Treg population.

Although previous studies have shown that CD206-mediated regulation of bvPLA2 on Treg cells plays an important role in the therapeutic effect of bvPLA2 on inflammatory diseases, the mechanisms underlying the action of bvPLA2 still remains poorly understood [7,8]. It has been known that bvPLA2 catalyzes the hydrolysis of the sn-2 ester bond of glycerophospholipids, leading to the release of fatty acids and lysophospholipids [7]. These by-products are further metabolized to a variety of lipid mediators, such as prostaglandins, leukotrienes, resolvins, platelet-activating factors, or lysophosphatidic acid [35]. These lipid mediators are recognized as important endogenous regulators of various biological processes, including cell proliferation, apoptosis, and inflammation. Therefore, future studies will be needed to investigate the role of glycerophospholipid-derived lipid mediators in the biological action of bvPLA2.

## 5. Conclusions

In conclusion, our data showed that administration of bvPLA2 ameliorated cholestatic liver injury and fibrosis in a murine model of cholestatic liver disease. The beneficial effects of bvPLA2 were associated with inhibition of hepatocyte apoptosis and inflammation. Although further studies are needed to elucidate detailed mechanisms, these results demonstrated for the first time the effect of bvPLA2 on cholestatic liver disease. Therefore, we propose that this enzyme could be a potential therapeutic option for treating the disease.

## Data Availability

Data are contained within the article.
